# How can precision health care contribute to healthy aging?

**DOI:** 10.1002/agm2.12333

**Published:** 2024-06-12

**Authors:** Jean‐Pierre Michel

**Affiliations:** ^1^ University of Geneva Geneva Switzerland; ^2^ French Academy of Medicine Paris France

## Abstract

The future of medicine will be closely linked to technological progress, to the great benefit of aging adults. Increasing knowledge in fields encompassing biology, physiology and functioning of the aging process, combined with the early detection of non‐clinically apparent but significant changes will make it possible to promote healthy aging.

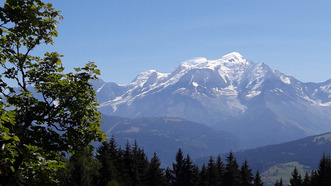

## 1 WHAT WE NEED TO KEEP IN MIND

The analysis of survival curves from the past two centuries clearly demonstrates that the decrease in child mortality is a key element explaining the amazing increase in life expectancy. It has now been proven that the increase in life expectancy worldwide, for both sexes, actually started back in the 19th century, rising from 52 years in the 1950s, to over 72 years today.

This fantastic achievement is linked to tremendous progress in medicine, particularly after the mid‐19th century, with the discovery and implementation of anesthesia (1850), insulin (1922), antibiotics (1928), and aspirin (1950), among others. Among these landmark discoveries, blood transfusion (1913) and vaccines (1955) are the two medical advancements that have saved the greatest number of lives, with an estimated billion lives saved by each. Beyond these purely numerical aspects of medical progress, technology began to enhance the impact of medicine on longevity, with the advent of cardio‐pulmonary resuscitation (1957), hemodialysis (1957) and pacemakers (1980), among many other advanced technologies. Since 2000, progress in bioengineering has included techniques and technologies such as robotic surgery (2003), nanotechnology (2003), big data (2010), brain and genetic mapping (2013)^1^ and indeed, artificial intelligence (2019).

This crucial progress easily explains the growth of the world's population, which will rise from less than 1 billion in 1800, to 6 billion in 1999, and probably reaching 10 billion by 2050. In this context, it is essential to stress that adults over 65 years will represent one fifth of the world population (i.e. 2 billion individuals). Bearing in mind that 80% of them will live in developing and moderately developed countries, it is evident that the health care challenges, in a constrained economy, will be extremely burdensome.

This is why it is urgent to enhance our understanding of the aging process, and to integrate it into a life‐course approach. Indeed, all the periods of life are important, as stressed by the 2015 WHO definition of healthy aging, namely: “Developing and maintaining functional ability to preserve well‐being until old age”.^2^ This shift in the definition of healthy aging clearly introduces the classical, but too often forgotten link between disease and function. These two are intricately linked, as illustrated by the coexistence of the ICD 11 (International Classification of Diseases)^3^ and the ICF (International Classification of Functions).^4^ This also testifies to the importance of controlling the disability process, which too often leads to dependency in old age, with all its personal and societal consequences.

## 2 ENLARGING THE CONCEPT OF PRECISION MEDICINE TO PRECISION HEALTH BEFORE FOCUSING ON PRECISION PREVENTION

Precision medicine, personalized medicine and high‐definition medicine are concepts that recently emerged hand in hand with the outstanding progress in biotechnology and human omics.^5^ Precision medicine is an innovative approach to tailoring disease prevention and treatment that considers differences in people's genes, environments, and lifestyles. In other words, precision medicine targets “the right treatment for the right patient at the right time”.^6^ This innovative approach is essential for a selected, and often quite limited population of individuals who would benefit from targeted, and generally quite costly treatments. The role of precision medicine is essential to fight against rare disease, for example by making it possible to discover gene therapy for Duchenne muscular dystrophy (for which FDA treatment approval was received in 2023). However, this type of highly specific medicine will certainly compound existing health care inequalities (in terms of access to, and cost of care), while ignoring the source of medical problems in the majority of the population (i.e. the burden of dependency due to population aging).^7^

These dangers have prompted the emergence of precision public health, which is an emerging concept to better predict and understand public health risks, and customize treatments for more specific and homogeneous subpopulations, often using new data, technologies, and methods.^7^

The application of these new technological tools will also enable the development of precision prevention, which will make it possible to rapidly identify people living with different levels of risk for a wider range of diseases. This new approach will be very useful and cost effective for future epidemiological research, and will help to better target specific interventions towards people at high risk of diseases or functional decline.

## 3 REVITALIZING PREVENTION CAMPAIGNS WITH NEW TECHNOLOGICAL DEVICES

The burden of diseases is sharply increasing with the aging of the world population. In sub‐Saharan countries, communicable diseases are currently the main cause of death. In all other parts of the world, non‐communicable conditions are more prevalent.^8^

Vaccinations are the best tool for preventing communicable diseases. Even before the COVID pandemic, the WHO affirmed that vaccines averted between 500 million and one billion deaths around the world.^9^ Moreover, as perfectly demonstrated during the COVID pandemic, technology can be leveraged to contribute quickly and efficiently to controlling the spread of emerging pathogens.

On the other hand, the most prevalent chronic diseases in the world, in decreasing order of frequency, are hypertension, hypercholesterolemia, arthritis, coronary heart disease, diabetes and chronic kidney disease, without forgetting dementia and psychiatric disorders.^10^ With aging, all these chronic pathologies accumulate, leading to multimorbidity, polypharmacy and ultimately, functional decline. In 2000, the WHO declared that eliminating modifiable exposures could potentially help to avoid 80% of all stroke, diabetes, and cardiovascular disease.^11^ Two decades later, studies confirmed that controlling 10 modifiable risk factors could reduce cerebrovascular disease by 90% and cancer by 42%.^12^

These facts plead in favor of the need to revitalize prevention campaigns with new technological devices, not only to prevent common diseases, but also to favor their early diagnosis, thus enabling quicker and more appropriate treatments. Below are three brief examples:

1. The first example is the life‐course approach to cardiovascular diseases. It is well known that genetics, birth conditions, family and environmental factors, as well as early life behavior can have a negative impact, and accelerate the development of atherosclerosis. All these young‐age factors will accumulate if the associated life habits (namely, poor nutrition, sedentary lifestyle, or smoking) are not controlled. After a few silent decades of life, acute events will occur at midlife.
  Nowadays, it is possible to receive alerts, to avoid missing out on early opportunities, using higher resolution pictures of cardiovascular risk factors, via wearable sensors and algorithms that favor interventions at asymptomatic or pre‐symptomatic stages of disease. In this way, the use of sensors can also evaluate the results of the proposed intervention. The use of innovative technologies will make it possible to choose the right treatment for specific subpopulations, with quicker dose adaptation, while also enabling the collection of exploitable health data, favoring the development of “long‐term personalized health”. The development of innumerable and diverse wearable sensors will enable general or specific analysis of cardiovascular, metabolic, respiratory, or mobility‐based bio‐signals.^13^ The sharp increase in the number of scientific papers about wearable or inert sensors for the analysis of health and aging proves that the methods described above represent the way forward for the future.^14^
2. The second example concerns the possible identification of the many midlife risk factors of cognitive decline, and the early diagnosis of dementia. New biological biomarkers, such as phospho‐Tau 217^15^ will soon be replaced by less invasive machine learning. Results demonstrate that text embedding, generated by the language model GPT‐3, can reliably be used to distinguish individuals with Alzheimer disease from healthy controls, and to infer the subject's cognitive testing score, solely on the basis of speech data.^16^
3. The third example concerns the early detection of pre‐frailty and frailty, which are known to precede disability. Numerous tools and applications already exist for the early identification of frailty. Among them is the WHO‐ICOPE, whose development around the world will be very useful.^17^ However, these tools need the involvement of trained professionals, managers to perform the complete clinical assessment, develop the diagnoses and target appropriate interventions. Much research is currently being devoted to comparing clinical assessment to the results obtained by passive wearable sensors included in clothes or home detection systems, combined with serious games, to facilitate the non‐invasive identification of pre‐frail or frail states.^18^ However, the most advanced technology using machine learning seems able to identify frailty based on 5 criteria namely age, cognition, large step distance, large step walking time and speed.^19^
  Moreover, technology combining machine learning and virtual reality makes it possible to facilitate frailty rehabilitation of populations at moderate or high risk.^20^

### TAKE HOME MESSAGE

The future of medicine will be closely linked to technological progress, to the great benefit of aging adults. Increasing knowledge in fields encompassing biology, physiology and functioning of the aging process, combined with the early detection of non‐clinically apparent but nevertheless meaningful changes will make it possible to promote healthy aging. At the same time, medicine will shift from its current reactive approach to a pro‐active approach, with earlier and effective interventions, which will benefit both individuals, and society as a whole.

## FUNDING INFORMATION

No funding.

## CONFLICT OF INTEREST STATEMENT

The author declares no conflicts of interest.
